# Initial Evaluation of the Internal Structure and Internal Consistency of the Context-Adapted Perceived Peer Motivational Climate Scale (CMI) Among Nursing Students Enrolled in a Clinical Simulation Program

**DOI:** 10.3390/bs16101932

**Published:** 2026-10-17

**Authors:** José Manuel García-Álvarez, Alfonso García-Sánchez

**Affiliations:** 1 Family and Community Medicine, Calasparra Health Center, 30420 Calasparra, Murcia, Spain; 2 Health Sciences PhD Program, Catholic University of Murcia (UCAM), Campus de los Jerónimos 135, 30107 Guadalupe, Murcia, Spain; 3 Anesthesiology and Critical Care, Vega Lorenzo Guirao Hospital, 30530 Cieza, Murcia, Spain; agarcia@ucam.edu; 4 Faculty of Nursing, Catholic University of Murcia (UCAM), Campus de los Jerónimos 135, 30107 Guadalupe, Murcia, Spain

**Keywords:** clinical simulation, nursing education, peer motivational climate, internal structure, internal consistency

## Abstract

**Background/Objectives**: Peer motivational climate may influence how students experience collaboration and learning in group-based educational settings, but its measurement properties have not been examined in nursing students enrolled in clinical simulation programs. The Perceived Peer Motivational Climate Scale (CMI) was originally developed in sport contexts, and its psychometric properties in this educational context have not been examined. Therefore, this study aimed to conduct an initial evaluation of the internal structure and internal consistency of a context-adapted CMI among nursing students enrolled in a clinical simulation program. **Methods**: A cross-sectional psychometric study was conducted with 395 fourth-year undergraduate nursing students at the Catholic University of Murcia (UCAM) who voluntarily agreed to participate, using a convenience sampling approach. The questionnaire was completed before the scheduled clinical simulation activities. Participants had previous experience with group work in clinical simulations, and the items referred to the peer group assigned to the upcoming simulation. However, it was not established whether all participants had previously interacted with those specific peers. Internal consistency was assessed using Cronbach’s alpha and McDonald’s omega coefficients for the task-involving and ego-involving motivational climate dimensions. The internal structure was examined using exploratory factor analysis (EFA) based on a polychoric correlation matrix, minimum residual extraction, and oblimin rotation, followed by confirmatory factor analysis (CFA) using the diagonally weighted least squares (DWLS) estimator for ordinal categorical variables. Both the EFA and CFA were conducted in the same sample. **Results**: The CMI demonstrated high internal consistency for both dimensions, with Cronbach’s alpha and McDonald’s omega coefficients of 0.939 for the task-involving dimension and 0.940 for the ego-involving dimension. Exploratory factor analysis was consistent with a two-factor structure, supported by parallel analysis, with items showing clear primary loadings and limited cross-loadings. Confirmatory factor analysis showed substantially better fit for the correlated two-factor model than for the one-factor model (estimator-adjusted Δχ^2^(1) = 126, *p* < 0.001; CFI = 0.991; TLI = 0.987; SRMR = 0.049), although the RMSEA remained elevated at 0.130 (95% CI 0.114 to 0.147). The two factors were positively correlated (r = 0.681). **Conclusions**: The CMI showed high estimated internal consistency and preliminary evidence consistent with a correlated two-factor structure in this sample of fourth-year nursing students enrolled in a clinical simulation program. These findings should be interpreted within the context of a single-institution convenience sample, the use of the same sample for EFA and CFA, and the absence of formal content-validity procedures in the target population. The study did not assess other sources of validity evidence, such as temporal stability, criterion-related validity, measurement invariance, or sensitivity to change. The generalizability of these findings beyond fourth-year nursing students at a single Spanish university participating in the specified simulation program remains uncertain. Further research using independent and more diverse samples, together with formal content-validity procedures and additional sources of validity evidence, is needed to replicate and extend these findings before broader use of the instrument can be considered.

## 1. Introduction

The training of healthcare professionals increasingly emphasizes collaborative and team-based approaches to address the complexity of healthcare systems and promote safe, patient-centered care (Babiker et al., 2014; Reeves et al., 2017; Rosen et al., 2018). In addition to technical competencies, effective healthcare teams require non-technical skills such as communication, leadership, coordination, shared decision-making, psychological safety, and teamwork, which contribute to effective team functioning and high-quality care (Anselmann et al., 2023; O’Donovan & McAuliffe, 2020; Rosen et al., 2018; Schmutz et al., 2019). Open communication, mutual trust, effective coordination, and psychological safety are also associated with team functioning, performance, and collective learning, highlighting their relevance to the education and development of future healthcare professionals (Anselmann et al., 2023; O’Donovan & McAuliffe, 2020; Reeves et al., 2017; Rosen et al., 2018; Schmutz et al., 2019).

Among these factors, motivational climate has attracted increasing attention due to its relationship with how individuals interpret success, regulate their behavior, and engage in learning and collaborative work (Nerstad et al., 2013; Deci et al., 2017; Ntoumanis & Biddle, 1999). From the perspective of Achievement Goal Theory, motivational climate refers to the social environment and patterns of cues that shape how success and achievement are defined, evaluated, and reinforced within a given context (Nerstad et al., 2013; Ntoumanis & Biddle, 1999). These perceptions are related not only to individual motivation but also to interpersonal processes, cooperation, and engagement with group goals (Deci & Ryan, 2008; Deci et al., 2017; Johnson & Johnson, 2009).

According to Achievement Goal Theory, a task-involving motivational climate is characterized by mastery, effort, cooperation, and personal improvement, whereas an ego-involving motivational climate emphasizes social comparison, competitiveness, and recognition based on outperforming others (Ntoumanis & Biddle, 1999). Self-Determination Theory provides a complementary contextual perspective on motivation, but it is not part of the measurement model examined in the present study. The CMI specifically assesses task-involving and ego-involving peer motivational climate dimensions derived from Achievement Goal Theory. These theoretical perspectives provide a basis for understanding motivational processes in collaborative learning environments, although the present study does not directly examine these processes or their outcomes (Deci & Ryan, 2008; Deci et al., 2017; Johnson & Johnson, 2009; Ntoumanis & Biddle, 1999). Previous research, largely conducted in sport and educational settings, suggests that supportive and collaborative motivational environments are associated with more adaptive motivational and interpersonal patterns, whereas competitive or ego-involving climates may be associated with different patterns of motivation and interaction (Deci et al., 2017; Johnson & Johnson, 2009; Keyko et al., 2016). However, the extent to which these findings apply to undergraduate nursing students participating in clinical simulation remains to be established.

Although early research on motivational climate focused primarily on the influence exerted by teachers, coaches, or supervisors (Ntoumanis & Biddle, 1999), subsequent studies indicated that peers also constitute a significant source of motivational influence (Ntoumanis & Vazou, 2005). Through their everyday interactions, peers help establish implicit norms regarding which behaviors are valued and what constitutes success within the group (Ntoumanis & Biddle, 1999; Ntoumanis & Vazou, 2005). This peer motivational climate becomes especially important in educational settings where learning depends on cooperation and continuous interaction among students (Johnson & Johnson, 2009; Ntoumanis & Vazou, 2005).

To assess this construct, Ntoumanis and Vazou (2005) developed the Peer Motivational Climate in Youth Sport Questionnaire (PeerMCYSQ), specifically designed to assess the motivational climate created by peers. Subsequently, Moreno-Murcia et al. (2006) developed the Spanish Perceived Peer Motivational Climate Scale (CMI) based on this instrument. The Spanish CMI comprises nine items assessing two dimensions: task-involving and ego-involving peer motivational climate. Previous research reported evidence supporting this two-factor structure and the internal consistency of the Spanish CMI in participants engaged in collective, non-competitive physical-sport activities (Moreno-Murcia et al., 2006). However, the instrument was developed and evaluated primarily in sport-related contexts, and its measurement properties have not been examined among nursing students enrolled in clinical simulation programs. This difference in context is relevant because the meaning and interpretation of peer interactions may depend on the characteristics and goals of the setting in which they occur.

Previous research, primarily conducted in sport and educational settings, has associated perceptions of a task-involving motivational climate with more adaptive motivational and interpersonal outcomes, whereas an ego-involving climate has been associated with different, and in some contexts less adaptive patterns of motivation and interaction (Deci & Ryan, 2008; Deci et al., 2017; Ntoumanis & Biddle, 1999; Ntoumanis & Vazou, 2005; Moreno-Murcia et al., 2006). Although these findings provide a theoretical basis for examining peer motivational climate in collaborative learning environments, their applicability to undergraduate nursing students enrolled in clinical simulation programs cannot be assumed and requires empirical investigation.

Clinical simulation is considered a widely used educational methodology for developing both technical and non-technical competencies in students and healthcare professionals (Cant & Cooper, 2010; Sezgin & Bektaş, 2023). Available systematic reviews and meta-analyses show that simulation can support the development of communication, leadership, teamwork, clinical decision-making, and patient safety-related competencies (Abildgren et al., 2022; Sezgin & Bektaş, 2023). In addition, recent studies suggest that simulation-based education is associated with higher levels of group cohesion among nursing students (García-Álvarez et al., 2026). These principles are reflected in the Healthcare Simulation Standards of Best Practice™, which provide international recommendations for the design, implementation, facilitation, and evaluation of simulation-based education (INACSL Standards Committee et al., 2025).

Clinical simulation activities require students to collaborate in managing complex clinical scenarios, exchanging information, coordinating their actions, and making collective decisions under conditions that may resemble aspects of real clinical practice (Abildgren et al., 2022; Cant & Cooper, 2010; INACSL Standards Committee et al., 2025; Sezgin & Bektaş, 2023). Following the simulation, debriefing is considered an important component of reflective learning because it allows participants to analyze individual and team performance, receive structured feedback, and integrate the experience with their prior knowledge (Duff et al., 2024; INACSL Standards Committee et al., 2025; Lee et al., 2020). In this regard, systematic reviews have shown that high-quality debriefing may support clinical reasoning, critical thinking, self-efficacy, and collaborative learning, and is therefore regarded as an essential component of contemporary simulation-based education (Duff et al., 2024; Lee et al., 2020). Within this context, peer interactions may be relevant to students’ engagement and to the collaborative learning process, particularly in educational environments characterized by sustained interaction and interdependence among students (Johnson & Johnson, 2009; Ntoumanis & Vazou, 2005).

One possible explanation for this relationship lies in psychological safety, defined as the shared belief that an environment allows individuals to take interpersonal risks without fear of negative consequences. Psychological safety is considered an important component of team learning and may be particularly relevant to effective clinical simulation environments (Edmondson, 1999; Edmondson & Lei, 2014; INACSL Standards Committee et al., 2025). It may facilitate open communication, knowledge sharing, and learning from mistakes, which are relevant to effective teamwork and learning in healthcare teams and clinical simulation (Edmondson, 1999; Edmondson & Lei, 2014; INACSL Standards Committee et al., 2025; Rosen et al., 2018). Accordingly, a task-involving motivational climate may be associated with more collaborative learning environments, whereas an ego-involving climate may be associated with different interpersonal and motivational processes that could hinder some aspects of collaboration (Deci et al., 2017; Nerstad et al., 2013; Ntoumanis & Biddle, 1999). However, psychological safety, motivation, and collaborative learning outcomes were not measured in the present study and are discussed here only as relevant concepts for future research.

According to the existing evidence, the use of this scale in healthcare education remains limited, and little is known about its internal structure and internal consistency among nursing students enrolled in clinical simulation programs. Given that this educational context differs from those in which the instrument was originally developed and previously evaluated, it is necessary to conduct an initial examination of whether its proposed two-factor structure is supported in this population before considering broader applications.

A context-adapted measure could provide a basis for future research examining peer motivational climate in clinical simulation. However, relationships with psychological safety, academic motivation, collaborative learning, teamwork, and non-technical competencies should be considered hypotheses for future research rather than outcomes established by the present study.

Accordingly, the objective of this study was to conduct an initial evaluation of the internal structure and internal consistency of a context-adapted Perceived Peer Motivational Climate Scale (CMI) among undergraduate nursing students enrolled in a clinical simulation program.

## 2. Materials and Methods

### 2.1. Ethics Statement

The study was conducted in accordance with the principles of the Declaration of Helsinki (World Medical Association, 2013) and was approved by the Ethics Committee of the Catholic University of Murcia (UCAM) (approval CE012310; approved on 26 January 2023). The ethical approval covered the study protocol and the context-adapted version of the instrument used in the present study. All students received clear and comprehensive information about the study. Before completing the questionnaire, participants were informed that participation was entirely voluntary, that written informed consent was required, and that they could withdraw at any time without any consequences. No incentives or compensation were provided for participation, and students who chose not to participate incurred no penalties. Participant privacy was protected by ensuring anonymity, as no personally identifiable information was collected.

### 2.2. Study Design

A cross-sectional psychometric study was conducted to provide an initial evaluation of the internal structure and internal consistency of the Perceived Peer Motivational Climate Scale (CMI), adapted for use in the context of clinical simulation with nursing students. Relevant concepts from the COSMIN framework were considered when evaluating these measurement properties (Prinsen et al., 2018), with its recommendations interpreted in the context of this educational and behavioral instrument.

### 2.3. Participants and Scope of Study

The study included fourth-year undergraduate nursing students at UCAM who were scheduled to take part in the clinical simulation program between October 2024 and July 2025. All fourth-year nursing students scheduled to participate in the program during the study period were considered eligible for participation. The study population was therefore restricted to fourth-year undergraduate nursing students enrolled at UCAM and scheduled to participate in the specified clinical simulation program during the study period.

The questionnaire was administered in Spanish, the language of instruction of the nursing program, and all participants were able to understand and complete the questionnaire in Spanish; no language-related difficulties were reported. All participants had previous exposure to clinical simulation as part of their undergraduate nursing training. Each student was allowed to complete the questionnaire only once, and repeated questionnaire responses were not permitted.

### 2.4. Sample Selection

The sample was selected using a non-probability convenience sampling method based on the student groups assigned by the UCAM Nursing Clinical Placement Unit. All eligible students within these assigned groups were invited to participate in the study.

The inclusion criteria were being a fourth-year undergraduate nursing student, being scheduled to participate in the clinical simulation program during the study period, and voluntarily agreeing to participate in the study. No specific age-based exclusion criterion was applied; eligibility was based on enrollment in the fourth year of the undergraduate nursing program. Participants who did not meet the eligibility criteria were excluded from the study. Participants with incomplete CMI responses would have been excluded from the complete-case analytical sample; however, no such exclusions were necessary because all 395 participants provided complete responses.

No formal a priori sample size calculation was performed. The available sample was considered in relation to the specific characteristics of the planned psychometric analyses, as sample-size requirements for factor analysis depend on characteristics such as the complexity of the factor structure, communalities, factor loadings, and the number of indicators rather than on a single participants-per-item rule (Costello & Osborne, 2005; DeVellis & Thorpe, 2021; Worthington & Whittaker, 2006). The CMI comprises nine ordered-categorical items representing two correlated factors, with five indicators for the task-involving dimension and four for the ego-involving dimension. The hypothesized model is relatively parsimonious, with a limited number of factor loadings, factor covariance, and residual parameters to be estimated. The original two-factor structure provided an a priori theoretical basis for the expected factor configuration, and the five response categories and sensitivity analyses using an alternative correlation matrix and extraction method were also considered.

Because individual simulation-group identifiers were not recorded, the potential effect of within-group dependence on the effective sample size could not be quantified. This limitation was considered when interpreting the precision and generalizability of the findings.

### 2.5. Measurement Instrument

The Perceived Peer Motivational Climate Scale (CMI), developed by Moreno-Murcia et al. (2006) based on the Peer Motivational Climate in Youth Sport Questionnaire developed by Ntoumanis and Vazou (2005), was used to assess students’ perceptions of the motivational climate generated by their peers. Moreno-Murcia et al. selected nine items from the original instrument to develop the Spanish CMI. The resulting nine-item CMI comprises two dimensions, task-involving climate (Items 1, 3, 5, 6, and 7) and ego-involving climate (Items 2, 4, 8, and 9), with responses rated on a five-point Likert-type scale ranging from 1 (never) to 5 (always). The task-involving items reflect complementary aspects of a peer climate characterized by cooperation, support, positive interpersonal experiences, and personal improvement: Item 1 refers to enjoyment of working with others; Items 3 and 6 reflect encouragement and support for peer improvement; Item 5 reflects positive experiences with peers; and Item 7 reflects freedom to express opinions within the group. In their study, Moreno-Murcia et al. reported evidence supporting the two-factor structure and internal consistency of the Spanish CMI in a sample of participants engaged in collective, non-competitive physical-sport activities. Because the instrument was originally developed in a sports context, its internal structure and internal consistency were re-evaluated in the present sample of nursing students participating in clinical simulation.

To adapt the instrument to the clinical simulation context, the two authors independently reviewed the Spanish version of the original items and proposed semantic and contextual adaptations for the target setting. The introductory stem was modified to refer specifically to the simulation group: “En el grupo que realizó la simulación clínica, los compañeros(as) …”. The questionnaire was administered before the scheduled simulation session, and the items were intended to refer to the peers assigned to the upcoming simulation. Although participants had previous experience with group work in clinical simulations, we did not establish whether they had previously interacted with all members of their assigned group or how they interpreted the introductory wording, which used past-tense language despite the timing of administration.

At the item level, Item 1 was modified from “Se divierten cuando practican con otros” to “Se divierten cuando trabajan con otros”, whereas the wording of Items 2–9 was retained. As the Spanish version of the instrument was already available, no translation or back-translation procedures were required. Any discrepancies between the authors’ proposals were resolved by consensus. No changes were made to the scale structure, number of items, response options, or the intended meaning of the constructs. Thus, the adaptation was limited to the contextual referent of the instrument and the wording of Item 1. The adapted version of the CMI used in the present study is shown in Table 1. The questionnaire was administered in Spanish, and the “Adapted Spanish version administered” column provides the exact wording used with participants. The English translations are provided for reporting purposes only and were not administered to participants.

However, no formal content-validity assessment involving experts or representatives of the target population was conducted before data collection. Therefore, the adaptation was considered a semantic and contextual adaptation rather than a formal evaluation of content validity.

### 2.6. Data Collection

Data were collected using a questionnaire comprising sociodemographic variables and the CMI adapted to the clinical simulation context. The questionnaire was completed before the scheduled clinical simulation activities. Students who chose to participate completed the questionnaire voluntarily after providing written informed consent. Clinical simulation activities in the program were organized in groups of 14–16 students.

The CMI items were intended to refer to the peer group assigned to the upcoming simulation activities. Although participants had previous experience with group work in clinical simulations, it was not established whether all participants had previously interacted with the specific peers in their assigned groups. Furthermore, the introductory stem used past-tense wording despite the questionnaire being administered before the scheduled simulation, and participants’ interpretation of this wording was not assessed. Individual group identifiers were not recorded in the study dataset; therefore, participants could not be retrospectively linked to their specific simulation groups.

### 2.7. Variables

The sociodemographic variables collected included university, age, gender, region of origin, previous academic qualification, and employment status. The primary study variables were the scores obtained on the individual items and two CMI dimensions (task-involving and ego-involving motivational climate). No total CMI score was interpreted in the present study.

### 2.8. Data Analysis

Statistical analyses were conducted using Jamovi (version 2.7.9.0) and R (version 4.5.0). Descriptive statistics and internal consistency analyses, including the estimation of Cronbach’s alpha and McDonald’s omega, were performed in Jamovi using the EFA module. The primary ordinal exploratory factor analysis (EFA) was conducted in R using the psych package. Polychoric correlations were estimated using psych::polychoric(), and the EFA was conducted using psych::fa() with minimum residual extraction (fm = “minres”) and oblimin rotation. Parallel analysis was conducted in R using the Pearson correlation matrix of the nine items to determine the number of factors to retain. A sensitivity EFA was conducted using the Pearson correlation matrix, maximum likelihood extraction, and oblimin rotation. Confidence intervals for Cronbach’s alpha and McDonald’s omega were estimated in R using 5000 nonparametric bootstrap resamples with replacement.

Confirmatory factor analyses (CFA), including the calculation of Composite Reliability (CR) and Average Variance Extracted (AVE), were conducted using the SEMLj module (version 1.2.8) in jamovi, which uses the lavaan package (version 0.6-19) as the underlying estimation framework. Given the ordinal nature of the response categories, the CFA models were estimated using the diagonally weighted least squares (DWLS) estimator with delta parameterization.

The heterotrait–monotrait ratio (HTMT) was calculated directly in R using polychoric correlations estimated with lavaan::lavCor(). Its confidence interval was obtained using 5000 bootstrap resamples.

Descriptive statistics were calculated first for all items, including the mean, standard deviation, median, interquartile range, minimum and maximum values, and response frequencies. Item skewness and kurtosis were also examined, together with the distribution of responses across the five response categories. The frequencies and percentages of responses across the five response categories were also examined to describe the distribution of responses and the extent to which each response category was used.

The internal consistency of each CMI dimension was evaluated using Cronbach’s alpha (α) and McDonald’s omega total (ω_t_). Alpha and omega coefficients of ≥0.70 were considered indicative of acceptable internal consistency, with higher values interpreted as stronger internal consistency. These thresholds were treated as general guidelines rather than definitive evidence of reliability. Corrected item–rest correlations and the effect of deleting each item within its respective dimension were also examined. Internal consistency was not interpreted as evidence of other forms of reliability.

The factorial structure of the instrument was assessed using complementary exploratory and confirmatory approaches. First, an exploratory factor analysis (EFA) was conducted using a polychoric correlation matrix, minimum residual extraction, and oblimin rotation, given the ordinal nature of the five-point response categories. The number of factors was initially assessed using parallel analysis based on the Pearson correlation matrix, supplemented by inspection of the scree plot, eigenvalues, and theoretical interpretability. Because the primary EFA was based on polychoric correlations, an additional parallel analysis using polychoric correlations was conducted as a sensitivity check. This analysis also supported the retention of two factors. As a further sensitivity analysis, a maximum likelihood EFA with oblimin rotation was also conducted to examine whether the factor solution was materially affected by the choice of estimation approach. Before factor extraction, the suitability of the Pearson correlation matrix used in the sensitivity EFA was evaluated using the Kaiser–Meyer–Olkin (KMO) measure of sampling adequacy and Bartlett’s test of sphericity. A KMO value ≥ 0.70 was considered indicative of adequate sampling. The EFA was considered exploratory, and the pattern matrix, communalities, and cross-loadings were examined to assess the interpretability of the factor solution. Standardized factor loadings of ≥0.40 were considered salient for interpretation, whereas loadings below 0.40 were considered weak. Cross-loadings were examined for substantial loadings on more than one factor, with a secondary loading ≥0.30 considered potentially indicative of insufficiently distinct factor membership. Communalities ≥ 0.40 were considered indicative of an item sharing an adequate proportion of variance with the retained factors. These values were used as descriptive guidelines to support interpretation of the factor solution rather than as automatic criteria for retaining or removing items.

Because the same participants contributed to both analyses, the subsequent confirmatory factor analysis (CFA) was considered an internal model check rather than an independent replication or cross-validation of the factor structure. The prespecified one-factor and correlated two-factor models were examined using CFA. Given the ordinal nature of the Likert-type items, the prespecified CFA models were estimated using the diagonally weighted least squares (DWLS) estimator for ordinal categorical variables, with delta parameterization. Model fit was evaluated using the scaled chi-square statistic (χ^2^), Comparative Fit Index (CFI), Tucker–Lewis Index (TLI), Standardized Root Mean Square Residual (SRMR), and Root Mean Square Error of Approximation (RMSEA), together with its 95% confidence interval. Fit indices were interpreted using commonly reported reference values, including CFI and TLI values around or above 0.90, and RMSEA and SRMR values around or below 0.08, while considering the overall pattern of fit indices and the characteristics of the model rather than relying on isolated cutoff values (Hu & Bentler, 1999; Kline, 2023). Because the DWLS estimator was used with ordinal data, RMSEA was interpreted in conjunction with the other fit indices rather than in isolation.

The standardized factor loadings, latent factor correlation and its confidence interval, and model convergence were also examined. Modification indices and residual correlations were reviewed for diagnostic purposes to identify potential sources of local misfit; however, no post hoc model modifications were introduced. In addition, Composite Reliability (CR) and Average Variance Extracted (AVE) were calculated for each dimension of the instrument based on the final CFA model. These coefficients were interpreted as model-based evidence of composite reliability and convergent validity rather than as comprehensive evidence of measurement validity. CR values ≥ 0.70 and AVE values ≥ 0.50 were considered commonly used reference values for evaluating model-based internal measurement quality and shared variance among indicators, respectively, following Fornell and Larcker (1981). The heterotrait–monotrait ratio (HTMT) was also calculated to provide additional model-based information regarding the distinctiveness of the two dimensions. An HTMT value below 0.85 was considered indicative of adequate discriminant validity, while a 95% bootstrap confidence interval that did not include 1.00 was considered additional evidence supporting discriminant validity. The HTMT confidence interval was estimated using 5000 bootstrap resamples.

Statistical significance was evaluated using a two-sided significance level of α = 0.05. All hypothesis tests were two-sided, and confidence intervals were reported at the 95% level.

No formal analysis protocol was preregistered before the data were examined. All analyses conducted as part of the present psychometric evaluation are reported in the manuscript and Supplementary Materials. No participants or CMI items were excluded after inspection of the data, and all participants with complete CMI responses were retained for analysis. The factor structure and CFA models were not modified post hoc to improve model fit.

## 3. Results

### 3.1. Sample Characteristics

The eligible cohort comprised 446 fourth-year nursing students. Of these, 395 participated in the study and were included in the analysis, corresponding to a participation rate of 88.6%, while 51 students declined participation. All 395 participants provided complete responses to the nine CMI items. Thus, there were no item-level missing data, no participants were excluded because of incomplete CMI responses, and no missing-data imputation was required. The final sample provided approximately 44 participants per item for the nine-item instrument. This ratio is reported descriptively and should not be interpreted as evidence of sample-size adequacy in itself.

The participants had a mean age of 23.65 years (SD = 5.74). Most participants were women (72.9%), were from the Region of Murcia (73.9%), had no previous academic qualification (82.0%), and were not employed while pursuing their nursing degree (89.9%).

### 3.2. Descriptive Statistics of the Items

Descriptive statistics and response distributions for the nine CMI items are presented in Table 2 and Table 3. All items had complete data (N = 395), with no missing responses. Mean item scores ranged from 2.89 to 3.26, and standard deviations ranged from 1.07 to 1.25. Median scores were 3 for all items, with an interquartile range of 2. Observed responses covered the full response range from 1 to 5 for all items. Skewness values ranged from −0.143 to 0.171, whereas kurtosis values ranged from −1.030 to −0.818. Response frequencies showed variability across all five response categories, with all categories represented across the nine items. The proportion of responses in the minimum category ranged from 2.5% to 15.7%, whereas the proportion in the maximum category ranged from 11.1% to 13.2%. These descriptive distributions showed no marked concentration of responses in either extreme category.

Descriptive statistics for the two CMI dimensions are presented in Table 4. Both subscale scores were calculated from complete responses from all 395 participants (N = 395 for each dimension). The task-involving climate dimension had a mean score of 16.00 (SD = 4.88), whereas the ego-involving climate dimension had a mean score of 11.70 (SD = 4.54). Median scores were 16 and 12, respectively, and the interquartile ranges were 8 and 7, respectively. The observed scores ranged from 5 to 25 for the task-involving dimension and from 4 to 20 for the ego-involving dimension. Skewness values were 0.091 and 0.033, and kurtosis values were −0.974 and −1.010 for the task-involving and ego-involving dimensions, respectively. The observed Pearson correlation between the two subscale scores was positive and statistically significant (r = 0.631, *p* < 0.001). The two dimensions were treated as separate scores, and no overall CMI score was calculated or interpreted.

### 3.3. Internal Consistency and Internal Structure

#### 3.3.1. Internal Consistency

The CMI showed high internal consistency estimates for both dimensions. Cronbach’s alpha was 0.939 for the task-involving motivational climate dimension and 0.940 for the ego-involving motivational climate dimension. The corresponding McDonald’s omega coefficients were 0.939 and 0.940, respectively (Table 5).

Item–rest correlations ranged from 0.806 to 0.857 for the task-involving dimension and from 0.837 to 0.872 for the ego-involving dimension. All item–rest correlations exceeded 0.30. No individual item deletion resulted in an improvement in the corresponding reliability coefficient, and all nine items were therefore retained for subsequent analyses.

These coefficients provide evidence of internal consistency for the two proposed dimensions; they do not, however, provide evidence regarding temporal stability, measurement invariance, or external validity.

#### 3.3.2. Internal Structure: Exploratory Factor Analysis

The nine items showed limited univariate skewness and kurtosis, with skewness ranging from −0.143 to 0.171 and kurtosis from −1.030 to −0.818 (Supplementary Table S1). The Kaiser–Meyer–Olkin measure of sampling adequacy was 0.915, with item-level measures ranging from 0.890 to 0.934. Bartlett’s test of sphericity was significant, χ^2^(36) = 3422, *p* < 0.001, supporting the suitability of the correlation matrix for factor analysis. Pearson and polychoric correlation matrices are provided in Supplementary Table S2.

An exploratory factor analysis (EFA) was conducted using a polychoric correlation matrix, minimum residual extraction, and oblimin rotation, given the ordinal nature of the response categories. Factor retention was determined primarily using parallel analysis, complemented by inspection of the scree plot and theoretical interpretability. The primary EFA was based on the polychoric correlation matrix. Parallel analysis based on the Pearson correlation matrix was used as complementary evidence for factor retention and supported a two-factor solution: the first two observed factor eigenvalues were 5.69 and 1.02, exceeding the corresponding simulated factor eigenvalues of 0.51 and 0.17, whereas the third observed factor eigenvalue (0.02) was lower than its simulated counterpart (0.12) (Table 6; Supplementary Table S3). The scree plot also showed a marked decline after the second factor, supporting the two-factor interpretation (Supplementary Figures S1 and S2). The polychoric-based parallel analysis also supported the retention of two factors, consistent with the factor-retention decision in the primary ordinal EFA (Supplementary Table S4).

The two-factor solution showed a pattern of item-factor associations broadly consistent with the proposed dimensions (Supplementary Table S5). Items 1, 3, 5, 6, and 7 loaded primarily on the task-involving factor, with pattern coefficients ranging from 0.704 to 0.991, whereas items 2, 4, 8, and 9 loaded primarily on the ego-involving factor, with coefficients ranging from 0.876 to 0.947. Cross-loadings ranged from −0.106 to 0.242. The correlation between the two factors was 0.626. Communalities ranged from 0.767 to 0.875 and uniquenesses from 0.125 to 0.233. The EFA converged without improper solutions or Heywood cases.

As a sensitivity analysis, a maximum likelihood EFA with oblimin rotation using the Pearson correlation matrix was also conducted. The same allocation of items to the two factors was obtained, with primary pattern coefficients ranging from 0.679 to 0.952 and absolute cross-loadings ≤ 0.235. Communalities ranged from 0.714 to 0.822, and the factor correlation was 0.621 (Supplementary Table S6).

Table 6 presents the factor-retention evidence and the main ordinal EFA results. The complete pattern matrix for the ordinal EFA is provided in Supplementary Table S5, while the pattern and structure matrices from the Pearson/maximum-likelihood sensitivity analysis are provided in Supplementary Tables S6 and S7, respectively.

#### 3.3.3. Internal Structure: Confirmatory Factor Analysis

The exploratory factor analysis identified a two-factor structure, which was subsequently examined using confirmatory factor analysis (CFA) in the same sample. The CFA was therefore considered an internal model check rather than an independent cross-validation. Both prespecified models were estimated: a one-factor model and a correlated two-factor model comprising task-involving and ego-involving dimensions.

The one-factor model showed mixed fit indices, with elevated SRMR and RMSEA values, with scaled χ^2^(27) = 676.99, CFI = 0.966, TLI = 0.954, SRMR = 0.160, and RMSEA = 0.247 (95% CI: 0.231 to 0.263). In contrast, the correlated two-factor model showed substantially improved fit, with scaled χ^2^(26) = 199.25, CFI = 0.991, TLI = 0.987, SRMR = 0.049, and RMSEA = 0.130 (95% CI: 0.114 to 0.147) (Table 7). An estimator-adjusted chi-square difference test indicated that the correlated two-factor model fit significantly better than the one-factor model, Δχ^2^(1) = 126, *p* < 0.001.

The correlated two-factor model converged successfully after 36 iterations, with no estimation warnings. Standardized factor loadings were high and statistically significant for all items, ranging from 0.900 to 0.925 for the task-involving factor and from 0.901 to 0.936 for the ego-involving factor (all *p* < 0.001). Standardized residual variances ranged from 0.145 to 0.191 for the task-involving items and from 0.123 to 0.189 for the ego-involving items (Table 8). Complete unstandardized and standardized parameter estimates, including standard errors, 95% confidence intervals, ordinal thresholds, residual variances, latent factor variances, and covariance estimates, are reported in Supplementary Table S8.

The standardized latent factor correlation was 0.681 (95% CI: 0.623 to 0.739, *p* < 0.001), indicating a substantial association between the task-involving and ego-involving dimensions.

Although the correlated two-factor model showed substantially better fit than the one-factor model, the fit indices did not provide uniformly satisfactory evidence of absolute fit. In particular, the RMSEA remained elevated, with a 95% confidence interval ranging from 0.114 to 0.147. Overall, the CFA results favored the correlated two-factor model over the unidimensional model, although the elevated RMSEA indicates that the adequacy of the absolute model fit remains uncertain.

#### 3.3.4. Model-Based Reliability and Additional Indicators

Additional model-based indicators are reported in Supplementary Table S9. Composite reliability (CR) was 0.960 for the task-involving dimension and 0.957 for the ego-involving dimension, while average variance extracted (AVE) was 0.828 and 0.846, respectively. The heterotrait–monotrait ratio (HTMT) between the two dimensions was 0.675, with a 95% bootstrap confidence interval ranging from 0.613 to 0.730.

These estimates provide additional model-based information on the internal measurement properties of the correlated two-factor model. The high CR values indicate high model-based composite reliability estimates, while the AVE values exceeded the conventional threshold of 0.50, indicating that, under the fitted model, each dimension accounted for a substantial proportion of variance in its indicators. The HTMT estimate was below the prespecified threshold of 0.85, and its 95% bootstrap confidence interval did not include 1.00, which is consistent with, but does not establish conclusively, discriminant separation between the two dimensions. These findings should nevertheless be interpreted as model-based evidence rather than as comprehensive evidence of convergent or discriminant validity, particularly because the analyses were conducted in a single sample and no external criterion measures were included.

## 4. Discussion

The findings provide preliminary evidence regarding the instrument’s internal structure and internal consistency in this educational context. The results supported a two-factor structure corresponding to task-involving and ego-involving motivational climate dimensions. This distinction is consistent with the broader theoretical conceptualization of motivational climate, in which social environments can emphasize task-related mastery or performance-related comparison (Ames, 1992; Ntoumanis & Biddle, 1999), and with self-determination theory, which emphasizes the role of social environments in shaping motivational processes (Deci & Ryan, 2008; Ryan & Deci, 2017). However, given the single-institution sample, the use of the same sample for EFA and CFA, and the absence of formal content-validity assessment in the target population, the findings should be considered an initial evaluation rather than evidence of comprehensive instrument validity.

The descriptive statistics indicated substantial coverage of the response scale, with all five response categories represented across the items and no marked concentration at the response extremes. The relatively balanced distributions and the absence of missing responses indicate that all response categories were used and that item-level missingness was minimal in this sample. However, these descriptive findings should not be interpreted as evidence of discriminative validity or of the instrument’s performance in distinguishing between groups of students. More broadly, psychometric evaluation requires evidence from multiple sources rather than relying on item distributions or internal consistency alone (Boateng et al., 2018; DeVellis & Thorpe, 2021).

The internal consistency estimates for the task-involving and ego-involving dimensions were high, with similar estimates obtained using Cronbach’s alpha and McDonald’s omega. These findings indicate high internal consistency estimates for the items within each dimension. The use of omega alongside alpha is consistent with recommendations that reliability assessment should not rely exclusively on Cronbach’s alpha (Dunn et al., 2014; Hayes & Coutts, 2020). Given the small number of items in each dimension (five and four items), the coefficients should nevertheless be interpreted alongside the item content and not as evidence that the instrument has been shown to possess all forms of reliability. In particular, temporal stability was not assessed, and high internal consistency does not by itself establish the absence of item redundancy or broader measurement reliability (DeVellis & Thorpe, 2021).

The observed reliability estimates were broadly consistent with those reported in previous applications of the CMI (Moreno-Murcia et al., 2006; Ntoumanis & Vazou, 2005), although direct comparison should be made cautiously because the present study involved a different population and educational context. The present adaptation was applied to nursing students participating in clinical simulation, whereas the original instrument was developed in the context of youth sport (Ntoumanis & Vazou, 2005) and subsequently examined in Spanish physical-sport participants (Moreno-Murcia et al., 2006). Both settings involve peer interaction, shared activities, feedback, and social comparison, which may provide contexts in which motivational climate is perceived through interpersonal processes. However, the present study did not directly examine whether these contextual features generate equivalent motivational processes, and therefore this explanation remains theoretical rather than an empirical finding.

Parallel analysis and the scree plot supported a two-factor solution, which was also in line with the theoretical structure of the instrument. This interpretation is consistent with methodological recommendations to consider empirical criteria together with theoretical considerations when determining the number of factors (Costello & Osborne, 2005). The primary EFA was conducted using a polychoric correlation matrix, minimum residual extraction, and oblimin rotation, given the ordinal response format. The resulting two-factor pattern was consistent with the theoretical structure of the instrument. A maximum-likelihood EFA based on the Pearson correlation matrix was conducted as a sensitivity analysis and yielded a similar pattern.

Because EFA and CFA were conducted in the same sample, these findings should not be interpreted as an independent replication of the factor structure. Rather, the CFA provided an internal check of the proposed factor structure in the same dataset. Independent replication in a separate sample would provide stronger evidence regarding the reproducibility and generalizability of the factor structure, as recommended in scale-development and validation research (Boateng et al., 2018; DeVellis & Thorpe, 2021).

The results of the confirmatory factor analysis provided evidence favoring the correlated two-factor model over the one-factor alternative. The two-factor model showed better CFI and TLI values and a lower SRMR than the one-factor model, and the nested model comparison also supported the two-factor model. At the same time, the fit indices did not provide uniformly satisfactory evidence of absolute model fit, particularly because the RMSEA remained elevated (RMSEA = 0.130, 95% CI: 0.114–0.147). Therefore, the CFA results should be interpreted as evidence of better relative fit for the two-factor model rather than as demonstrating unequivocally good overall model fit. The interpretation of model fit should consider multiple indices and the characteristics of the model rather than relying on isolated cutoff values (Hu & Bentler, 1999; Kline, 2023). In addition, the interpretation of RMSEA warrants caution in models with small degrees of freedom, as it may be sensitive to model complexity (Kenny et al., 2015). The estimator-adjusted chi-square difference test also indicated a statistically significant difference between the models, Δχ^2^(1) = 126, *p* < 0.001.

The standardized factor loadings were high across all items, indicating substantial relationships between the observed items and their respective latent dimensions. These loadings provide evidence regarding the internal measurement model but should not be interpreted as evidence of external validity or criterion-related validity (Boateng et al., 2018; DeVellis & Thorpe, 2021). The composite reliability (CR) and average variance extracted (AVE) values provided additional model-based information about the measurement model, with high CR and AVE values for both dimensions. These indicators provide information about internal measurement quality and shared variance among indicators, but they should not be interpreted as comprehensive evidence of convergent validity (Fornell & Larcker, 1981; Boateng et al., 2018). Similarly, the HTMT estimate provided supplementary information regarding the relationship between the two constructs, but a single HTMT value does not constitute comprehensive evidence of discriminant validity. Broader construct validation would require relationships with theoretically relevant external measures and, where appropriate, tests of known-groups or criterion-related validity (Boateng et al., 2018; DeVellis & Thorpe, 2021).

The positive association between the two observed subscale scores (r = 0.631, *p* < 0.001) and the latent factor correlation (r = 0.681) suggests that the two dimensions were meaningfully related while remaining conceptually distinguishable. This finding is compatible with motivational climate models in which different motivational emphases can coexist within the same social environment rather than necessarily representing mutually exclusive conditions (Ames, 1992; Ntoumanis & Biddle, 1999; Ntoumanis & Vazou, 2005). Higher scores on the ego-involving dimension should not necessarily be interpreted as indicating maladaptive or undesirable peer functioning. In the present context, the coexistence of task-involving and ego-involving perceptions should not be interpreted as necessarily indicating opposite forms of group functioning. Achievement-goal theory distinguishes between different motivational emphases without requiring one dimension to be interpreted as inherently negative or undesirable (Ames, 1992; Nicholls, 1989). The present study did not examine whether higher ego-involving scores were associated with beneficial or adverse educational outcomes. Consequently, the two dimensions should be interpreted as distinct perceptions of the peer motivational environment rather than as inherently positive and negative forms of group functioning.

The relevance of studying peer motivational climate in clinical simulation can be considered in the broader context of teamwork and collaborative learning in healthcare education. Teamwork is an important component of healthcare practice, and systematic evidence has linked teamwork processes with performance in healthcare teams (Schmutz et al., 2019). Reviews and conceptual work have also highlighted teamwork as an important component of safer and higher-quality healthcare (Rosen et al., 2018), while core teamwork competencies include coordination, communication, mutual support, and shared mental models (Salas et al., 2005). Interprofessional collaboration has likewise been examined as a means of improving professional practice and healthcare outcomes (Reeves et al., 2017).

Clinical simulation provides an educational environment in which students interact repeatedly, undertake shared tasks, receive feedback, and participate in structured reflection. Simulation-based learning has been widely examined in nursing education (Cant & Cooper, 2010), and systematic reviews have reported evidence regarding the effects of simulation-based education on teamwork and communication among healthcare students (Sezgin & Bektaş, 2023) and on human-factor skills in healthcare teams (Abildgren et al., 2022). However, the present study did not directly assess whether either type of motivational climate affects communication, participation, psychological safety, debriefing, teamwork, or learning outcomes. Therefore, potential relationships between perceived peer motivational climate and these processes remain hypotheses for future research rather than conclusions supported by the present data.

Psychological safety has been described as a condition that facilitates interpersonal risk-taking and learning behavior within teams (Edmondson, 1999), and subsequent work has examined its development and relevance in organizational and healthcare contexts (Edmondson & Lei, 2014; O’Donovan & McAuliffe, 2020). However, psychological safety was not measured in the present study, and no direct relationship between CMI scores and psychological safety can therefore be inferred. Similarly, the literature on nursing work engagement and burnout indicates that professional and organizational environments may be relevant to nurses’ professional experiences. Work engagement has been examined as an important dimension of professional nursing practice (Keyko et al., 2016), while burnout research has highlighted the importance of the work environment and the relationship between individuals and their occupational context (Maslach & Leiter, 2016). These outcomes were outside the scope of the present study and should not be considered consequences of the motivational climate measured by the CMI.

From an educational perspective, the adapted CMI may provide a basis for further research on nursing students’ perceptions of peer motivational climate in the context of clinical simulation programs. Recent work examining clinical simulation and group cohesion also suggests that simulation can be a relevant context for studying group-related processes among nursing students (García-Álvarez et al., 2026). However, the present findings do not establish that CMI scores can be used to identify student groups requiring intervention or to evaluate the effectiveness of interventions. Future research could examine whether perceived peer motivational climate varies according to simulation design, facilitation approach, group composition, or debriefing method, and whether these perceptions are associated with objectively measured educational outcomes. This would be particularly relevant because simulation facilitation and debriefing are recognized components of simulation-based education, with current standards emphasizing facilitation processes and structured approaches to supporting learning (INACSL Standards Committee et al., 2025). Systematic reviews have also examined how different debriefing approaches relate to learning outcomes in healthcare and nursing simulation (Duff et al., 2024; Lee et al., 2020).

Several limitations should be considered. The use of a convenience sample from a single institution limits the generalizability of the findings. The results should therefore not be assumed to generalize to students from other academic years, other nursing programs, other health-science disciplines, healthcare professionals, other institutions, languages, cultures, or simulation formats. Replication in independent samples from these contexts would be needed to determine whether the observed structure is reproducible across populations and educational settings.

The questionnaire was completed before the scheduled clinical simulation session, and its introductory stem referred to the peer group assigned to the upcoming simulation. Although participants had previous experience with group work in clinical simulations, it was not established whether they had previously interacted with all members of their assigned group. Consequently, it remains uncertain whether participants had sufficient prior experience with the specific peers to provide informed perceptions of that group’s motivational climate. This limitation should be considered when interpreting the findings, as responses may not have been based on direct previous interactions with all members of the assigned group. Future studies should clearly specify the peer group participants are expected to evaluate and establish whether they have had sufficient prior interaction with those peers.

In addition, participants were recruited through pre-existing student groups, and individual group identifiers were not available. Therefore, potential within-group clustering could not be assessed using intraclass correlations or accounted for through multilevel or cluster-adjusted analyses. Consequently, the analyses treated the 395 observations as independent, and potential within-group dependence may have affected standard errors and factor estimates.

All 395 participants provided complete responses to the nine CMI items, and no missing-data imputation was required. However, it was not possible to determine whether students who declined participation differed systematically from those who participated, which may limit the representativeness of the sample. The study also did not include a formal content-validity assessment involving the target population. This is particularly relevant because the CMI was originally developed in a sports context (Ntoumanis & Vazou, 2005; Moreno-Murcia et al., 2006) and was adapted for clinical simulation. Although two authors independently reviewed the adapted items and resolved discrepancies by consensus, no formal assessment involving experts or representatives of the target population, cognitive interviews, or pilot testing was conducted. Therefore, evidence regarding the relevance, comprehensiveness, and comprehensibility of the adapted items remains limited. Future studies should include formal content-validity assessment with experts and representatives of the target population, consistent with recommendations for scale development and validation (Worthington & Whittaker, 2006; DeVellis & Thorpe, 2021; Boateng et al., 2018).

EFA and CFA were conducted using the same sample. Although retaining the full sample increased statistical precision, the CFA cannot be considered an independent cross-validation. Independent replication in separate samples would provide stronger evidence regarding the reproducibility and generalizability of the observed factor structure. In addition, the study was not conducted under a prospectively registered or otherwise publicly available a priori analysis protocol. Although the analytical procedures were designed to evaluate the internal structure and internal consistency of the adapted CMI, the absence of a prespecified protocol reduces transparency regarding which analytical decisions were established before examining the data. Future studies should consider prospectively specifying the analytical plan, including factor-retention criteria, model specifications, estimators, and primary psychometric outcomes.

The cross-sectional design did not allow assessment of temporal stability or measurement stability over time. Test–retest reliability and other indicators of measurement stability should therefore be examined in future studies. Longitudinal designs could also be used to investigate whether perceptions of peer motivational climate change across simulation experiences and whether the instrument is sensitive to such changes. Measurement invariance across relevant groups should also be examined when sufficient samples are available.

The study did not specify or test a priori hypotheses concerning associations between CMI scores and external constructs. No external measures of theoretically related or distinct constructs were included, and therefore the study could not evaluate convergent, discriminant, criterion-related, or known-groups validity. The CR, AVE, and HTMT estimates provided supplementary model-based information but do not replace hypothesis-based testing of relationships with external variables. Future studies should prespecify theoretically grounded hypotheses and examine them using appropriate external measures. Because psychological safety, teamwork, communication, learning engagement, non-technical competence, academic performance, and patient safety were not measured in the present study, associations or causal relationships between peer motivational climate and these outcomes cannot be inferred.

The present study focused primarily on internal structure and internal consistency. Although CR, AVE, and HTMT provided supplementary model-based information regarding the measurement properties of the two-factor model, these indicators do not constitute comprehensive evidence of convergent or discriminant validity. In addition, cross-cultural validity was not assessed. A comprehensive body of validity evidence requires information from multiple sources and should not be based solely on structural analyses and internal consistency coefficients (Prinsen et al., 2018; Mokkink et al., 2018; Boateng et al., 2018).

Overall, the findings were consistent with the two-factor structure previously described for the CMI (Ntoumanis & Vazou, 2005; Moreno-Murcia et al., 2006). The present study extends the examination of the instrument to nursing students participating in clinical simulation, providing preliminary evidence regarding its internal structure and internal consistency in this context rather than evidence that the instrument has been comprehensively validated for use across health sciences students or clinical professionals.

The adapted CMI may therefore provide a useful starting point for research on peer motivational climate in healthcare education. Further work should focus on independent replication of the factor structure, formal content-validity assessment, measurement invariance, test–retest reliability, measurement error, and hypothesis-based examination of relationships with external educational outcomes. These studies could help determine whether perceived peer motivational climate is systematically related to teamwork, communication, psychological safety, learning, non-technical skills, clinical performance, or patient-safety-related outcomes, none of which were directly measured in the present study.

## 5. Conclusions

The adapted Perceived Peer Motivational Climate Scale (CMI) showed high estimated internal consistency and preliminary evidence consistent with a correlated two-factor interpretation among nursing students enrolled in a clinical simulation program. The findings should be interpreted as an initial evaluation of the scale’s internal structure and internal consistency in this sample rather than as evidence of comprehensive validity.

Further evaluation in independent samples is needed to determine whether the two-factor interpretation is reproducible and to provide additional evidence regarding the measurement properties of the adapted CMI. Formal assessment of content validity, measurement invariance, test–retest reliability, and relationships with external constructs should also be considered in future studies.

## Figures and Tables

**Table 1:** Adapted Version of the Perceived Peer Motivational Climate Scale (CMI) for Clinical Simulation.

Item	Original Spanish Version	Adapted Spanish Version Administered	English Translation
1	Se divierten cuando practican con otros.	Se divierten cuando trabajan con otros.	They enjoy working with others.
2	Quieren ser los mejores del grupo.	Quieren ser los mejores del grupo.	They want to be the best in the group.
3	Animan a los compañeros a no abandonar.	Animan a los compañeros a no abandonar.	They encourage their peers not to give up.
4	Se animan a ser mejor que los otros.	Se animan a ser mejor que los otros.	They encourage one another to outperform others.
5	Se sienten bien con los compañeros.	Se sienten bien con los compañeros.	They feel good with their peers.
6	Dan consejos a los compañeros para ayudarles a progresar.	Dan consejos a los compañeros para ayudarles a progresar.	They give their peers advice to help them improve.
7	Se sienten libres de expresar sus opiniones.	Se sienten libres de expresar sus opiniones.	They feel free to express their opinions.
8	Se comparan unos con otros.	Se comparan unos con otros.	They compare themselves with each other.
9	Intentan hacerlo mejor que los demás.	Intentan hacerlo mejor que los demás.	They try to do better than the others.

Note. The adapted questionnaire was preceded by the following introductory stem: “En el grupo que realizó la simulación clínica, los compañeros(as) …” (“In the group that carried out the clinical simulation, the peers …”). Responses were rated on a five-point scale (1 = never; 5 = always). Items 1, 3, 5, 6, and 7 correspond to the task-involving climate dimension, whereas Items 2, 4, 8, and 9 correspond to the ego-involving climate dimension. The “Adapted Spanish version administered” column reproduces the exact wording presented to participants. Bold text indicates wording modified from the original Spanish version. The English translations are provided for reporting purposes only and were not administered to participants. Adapted from Moreno-Murcia et al. (2006).

**Table 2:** Frequency and percentage of responses across the five response categories for each CMI item.

Item	1, n (%)	2, n (%)	3, n (%)	4, n (%)	5, n (%)
1	22 (5.6)	116 (29.4)	120 (30.4)	88 (22.3)	49 (12.4)
2	56 (14.2)	104 (26.3)	97 (24.6)	94 (23.8)	44 (11.1)
3	18 (4.6)	93 (23.5)	103 (26.1)	129 (32.7)	52 (13.2)
4	54 (13.7)	93 (23.5)	112 (28.4)	89 (22.5)	47 (11.9)
5	10 (2.5)	107 (27.1)	100 (25.3)	128 (32.4)	50 (12.7)
6	16 (4.1)	106 (26.8)	108 (27.3)	113 (28.6)	52 (13.2)
7	13 (3.3)	104 (26.3)	119 (30.1)	109 (27.6)	50 (12.7)
8	57 (14.4)	111 (28.1)	92 (23.3)	87 (22.0)	48 (12.2)
9	62 (15.7)	86 (21.8)	119 (30.1)	81 (20.5)	47 (11.9)

Note. CMI = Perceived Peer Motivational Climate Scale. Response categories ranged from 1 to 5. Percentages are based on the total sample (N = 395). No missing responses were observed.

**Table 3:** Descriptive statistics and distributional characteristics of the CMI items.

Item	N	Mean	SD	Median	IQR	Range	Min.	Max.	Skewness	Kurtosis
1	395	3.07	1.11	3	2	1–5	1	5	0.171	−0.818
2	395	2.91	1.23	3	2	1–5	1	5	0.066	−1.000
3	395	3.26	1.10	3	2	1–5	1	5	−0.143	−0.855
4	395	2.95	1.22	3	2	1–5	1	5	0.028	−0.927
5	395	3.26	1.07	3	2	1–5	1	5	−0.021	−0.986
6	395	3.20	1.10	3	2	1–5	1	5	0.013	−0.920
7	395	3.20	1.07	3	2	1–5	1	5	0.059	−0.862
8	395	2.89	1.25	3	2	1–5	1	5	0.132	−1.030
9	395	2.91	1.23	3	2	1–5	1	5	0.047	−0.916

Note. CMI = Perceived Peer Motivational Climate Scale; SD = standard deviation; IQR = interquartile range. No missing responses were observed.

**Table 4:** Descriptive statistics and correlation between the two CMI dimension scores.

Subscale	N	Mean	SD	Median	IQR	Observed Range	Skewness	Kurtosis
Task-involving climate (CT)	395	16.00	4.88	16	8	5–25	0.091	−0.974
Ego-involving climate (CE)	395	11.70	4.54	12	7	4–20	0.033	−1.010

Correlation between subscale scores: (CT − CE): *r* = 0.631, *p* < 0.001. Note: CMI = Perceived Peer Motivational Climate Scale; CT = task-involving climate; CE = ego-involving climate; IQR = interquartile range. Dimension scores were calculated as the sum of the corresponding item responses. The correlation represents the Pearson correlation between the two subscale scores. No overall CMI score was calculated or interpreted.

**Table 5:** Internal consistency of the adapted Perceived Peer Motivational Climate Scale (CMI).

Scale	No. of Items	Cronbach’s α (95% CI)	McDonald’s ω_t_ (95% CI)
Task-involving motivational climate	5	0.939 (0.929 to 0.948)	0.939 (0.929 to 0.948)
Ego-involving motivational climate	4	0.940 (0.928 to 0.949)	0.940 (0.929 to 0.949)

Note. α = Cronbach’s alpha; ω_t_ = McDonald’s omega total. The 95% confidence intervals were estimated using 5000 nonparametric bootstrap resamples with replacement. Item–rest correlations ranged from 0.806 to 0.857 for the task-involving dimension and from 0.837 to 0.872 for the ego-involving dimension. No individual item removal increased the corresponding reliability coefficient, and all nine items were retained.

**Table 6:** Exploratory factor analysis: factor-retention evidence and main ordinal EFA results.

Item	Pattern: Task	Pattern: Ego	h^2^	u^2^
1	0.704	0.242	0.767	0.233
2	0.027	0.904	0.850	0.150
3	0.774	0.200	0.833	0.167
4	0.008	0.918	0.852	0.149
5	0.888	0.056	0.855	0.145
6	0.956	−0.077	0.827	0.173
7	0.991	−0.106	0.861	0.139
8	−0.019	0.947	0.875	0.125
9	0.032	0.876	0.803	0.197
Factor retention (parallel analysis)				
Factor	Observed eigenvalue	Simulated eigenvalue		Retained
1	5.69	0.51		Yes
2	1.02	0.17		Yes
3	0.02	0.12		No

Note. Pattern coefficients are based on minimum residual extraction with oblimin rotation using the polychoric correlation matrix. h^2^ = communality; u^2^ = uniqueness. All pattern coefficients are reported without suppression; therefore, no threshold was applied to replace small coefficients with dashes. The correlation between the two factors was *r* = 0.626. Factor retention was based primarily on parallel analysis, retaining factors whose observed eigenvalues exceeded the corresponding simulated eigenvalues, complemented by scree-plot inspection and theoretical interpretability.

**Table 7:** Confirmatory factor analysis of the CMI.

Fit Index	One-Factor	Correlated Two-Factor
χ^2^	676.99	199.25
df	27	26
CFI	0.966	0.991
TLI	0.954	0.987
SRMR	0.160	0.049
RMSEA	0.247	0.130
95% CI for RMSEA	0.231 to 0.263	0.114 to 0.147
Estimator-adjusted Δχ^2^	—	126
Δdf	—	1
*p*	—	<0.001

Note. CFI = comparative fit index; TLI = Tucker–Lewis index; SRMR = standardized root mean square residual; RMSEA = root mean square error of approximation; CI = confidence interval; Δχ^2^ = estimator-adjusted chi-square difference between the nested models. Both models were estimated using DWLS with robust standard errors and a mean-adjusted scaled-and-shifted test statistic, treating the nine CMI items as ordered categorical variables. The reported χ^2^ values and fit indices correspond to the scaled solution. The RMSEA confidence intervals are 95% CIs. The correlated two-factor model comprised task-involving and ego-involving factors.

**Table 8:** Standardized factor loadings and residual variances of the correlated two-factor CFA of the CMI.

Factor	Item	Standardized Loading (β)	95% CI	Residual Variance
Task-involving	1	0.900	0.874 to 0.927	0.189
	3	0.925	0.906 to 0.944	0.145
	5	0.916	0.897 to 0.936	0.161
	6	0.900	0.875 to 0.924	0.191
	7	0.910	0.887 to 0.933	0.172
Ego-involving	2	0.924	0.899 to 0.948	0.147
	4	0.919	0.898 to 0.940	0.156
	8	0.936	0.916 to 0.956	0.123
	9	0.901	0.875 to 0.926	0.189

Note. β = standardized factor loading; CI = confidence interval. Residual variances are standardized residual variances. The 95% CIs were calculated using model-based standard errors.

## Data Availability

The original contributions presented in this study are included in the Supplementary Materials. Further inquiries can be directed to the corresponding author.

## Supplementary Materials

The following supporting information can be downloaded at: https://www.mdpi.com/article/10.3390/bs16101932/s1.
