# Supplementary material for: Initial Evaluation of the Internal Structure and Internal Consistency of the Context-Adapted Perceived Peer Motivational Climate Scale (CMI) Among Nursing Students Enrolled in a Clinical Simulation Program
Source: Behav Sci (Basel). 2026 Oct 17;16(10):1932. doi: 10.3390/bs16101932 (PMC13701605; doi:10.3390/bs16101932)
Supplement: Supplementary file 1 [file behavsci-16-01932-s001.zip › Supplementary_File_R_Code_Jamovi_Rj_Editor.pdf]

SUPPLEMENTARY FILE S2. R CODE FOR JAMOVI Rj EDITOR

Study: Initial Psychometric Evaluation of the Perceived Peer Motivational Climate Scale (CMI)

This file contains the R code used in the Jamovi Rj Editor for the ordinal exploratory factor analysis (EFA), parallel analyses, reliability estimates and bootstrap confidence intervals, confirmatory factor analyses (CFA), composite reliability (CR), average variance extracted (AVE), and the heterotrait-monotrait ratio (HTMT).

----
1. ORDINAL EXPLORATORY FACTOR ANALYSIS USING POLYCHORIC CORRELATIONS

Ordinal exploratory factor analysis was conducted in R using the psych package. Polychoric correlations were estimated using psych::polychoric(), and the EFA was conducted using psych::fa() with minimum residual extraction (fm = "minres") and oblimin rotation.

```
library(psych)

# Select the nine CMI items
datos <- data[, c("I1", "I2", "I3", "I4", "I5",
                  "I6", "I7", "I8", "I9")]

# Calculate the polychoric correlation matrix
poly <- psych::polychoric(datos)

# Perform the two-factor ordinal EFA
efa_ordinal <- psych::fa(
  poly$rho,
  nfactors = 2,
  rotate = "oblimin",
  fm = "minres",
  n.obs = nrow(datos)
)

# Display the results
print(efa_ordinal, cut = 0, sort = FALSE)
```

----
2. PARALLEL ANALYSIS USING PEARSON CORRELATIONS

Parallel analysis was conducted using the Pearson correlation matrix of the nine items to help determine the number of factors to retain.

```
library(psych)

# Select the nine CMI items
datos <- data[, c("I1", "I2", "I3", "I4", "I5",
                  "I6", "I7", "I8", "I9")]

# Ensure that all items are numeric
datos[] <- lapply(datos, function(x) as.numeric(as.character(x)))
```

```
# Conduct parallel analysis using Pearson correlations
set.seed(2026)

pa_pearson <- psych::fa.parallel(
  datos,
  fa = "fa",
  fm = "minres",
  cor = "cor",
  n.iter = 1000,
  quant = 0.95,
  plot = FALSE
)

print(pa_pearson)
```

---

3. PARALLEL ANALYSIS USING POLYCHORIC CORRELATIONS

Parallel analysis was also conducted using polychoric correlations.

```
library(psych)

# Select the nine CMI items
datos <- data[, c("I1", "I2", "I3", "I4", "I5",
                  "I6", "I7", "I8", "I9")]

# Conduct parallel analysis using polychoric correlations
set.seed(2026)

pa_poly <- psych::fa.parallel(
  datos,
  fa = "fa",
  fm = "minres",
  cor = "poly",
  n.iter = 1000,
  quant = 0.95,
  plot = TRUE
)

print(pa_poly)
```

---

4. BOOTSTRAP CONFIDENCE INTERVALS FOR CRONBACH'S ALPHA AND MCDONALD'S OMEGA

Confidence intervals for Cronbach's alpha and McDonald's omega were estimated using 5,000 nonparametric bootstrap resamples with replacement.

```
library(psych)

# Select the items and preserve their original 1-5 scores
items <- c("I1", "I2", "I3", "I4", "I5",
           "I6", "I7", "I8", "I9")

datos <- data[, items, drop = FALSE]
```

```r
datos[] <- lapply(datos, function(x) {
  as.numeric(as.character(x))
})

# Define the Task and Ego subscales
task <- datos[, c("I1", "I3", "I5", "I6", "I7")]
ego  <- datos[, c("I2", "I4", "I8", "I9")]

# Calculate the reliability coefficients
fiabilidad <- function(x) {
  c(
    alpha = psych::alpha(x, warnings = FALSE)$total$raw_alpha,
    omega = suppressWarnings(
      psych::omega(x, nfactors = 1, plot = FALSE)$omega.tot
    )
  )
}

# Estimate bootstrap confidence intervals
bootstrap <- function(x, B = 5000) {
  original <- fiabilidad(x)
  resultados <- matrix(NA_real_, nrow = B, ncol = 2)
  colnames(resultados) <- c("alpha", "omega")

  for (b in seq_len(B)) {
    indices <- sample(seq_len(nrow(x)), replace = TRUE)
    xb <- x[indices, , drop = FALSE]

    resultados[b, ] <- tryCatch(
      fiabilidad(xb),
      error = function(e) c(alpha = NA_real_, omega = NA_real_)
    )
  }

  salida <- t(sapply(1:2, function(j) {
    valores <- resultados[, j]
    valores <- valores[is.finite(valores)]

    c(
      Estimate = original[j],
      CI_lower = quantile(valores, 0.025, names = FALSE),
      CI_upper = quantile(valores, 0.975, names = FALSE),
      Valid_resamples = length(valores)
    )
  }))

  rownames(salida) <- c("Cronbach_alpha", "McDonald_omega")
  salida
}

set.seed(12345)

cat("TASK SUBSCALE\n")
print(bootstrap(task))

cat("\nEGO SUBSCALE\n")
print(bootstrap(ego))
```

----
5. CONFIRMATORY FACTOR ANALYSIS: ONE-FACTOR AND CORRELATED TWO-FACTOR MODELS

Confirmatory factor analyses were conducted using the lavaan package. The nine items were treated as ordinal, and the DWLS estimator, robust standard errors, the scaled-and-shifted test statistic, and delta parameterization were specified.

```
library(lavaan)

# Define the nine ordinal items
items <- c("I1", "I2", "I3", "I4", "I5", "I6", "I7", "I8", "I9")

# Check that all items are present in the dataset
stopifnot(all(items %in% names(data)))

# Specify the one-factor model
modelo_1f <- '
  CMI =~ I1 + I2 + I3 + I4 + I5 + I6 + I7 + I8 + I9
'

# Specify the correlated two-factor model
modelo_2f <- '
  Task =~ I1 + I3 + I5 + I6 + I7
  Ego  =~ I2 + I4 + I8 + I9
'

# Fit the one-factor model
fit_1f <- cfa(
  model = modelo_1f,
  data = data,
  ordered = items,
  estimator = "DWLS",
  se = "robust",
  test = "scaled.shifted",
  parameterization = "delta"
)

# Fit the correlated two-factor model
fit_2f <- cfa(
  model = modelo_2f,
  data = data,
  ordered = items,
  estimator = "DWLS",
  se = "robust",
  test = "scaled.shifted",
  parameterization = "delta"
)

# Extract fit indices for each model
indices_ajuste <- function(modelo) {
  fitMeasures(
    modelo,
    c(
```

```
      "chisq",
      "df",
      "pvalue",
      "chisq.scaled",
      "df.scaled",
      "pvalue.scaled",
      "cfi.scaled",
      "tli.scaled",
      "rmsea.scaled",
      "rmsea.ci.lower.scaled",
      "rmsea.ci.upper.scaled",
      "srmr"
    )
  )
}

cat("\nONE-FACTOR MODEL\n")
print(round(indices_ajuste(fit_1f), 3))

cat("\nCORRELATED TWO-FACTOR MODEL\n")
print(round(indices_ajuste(fit_2f), 3))

# Extract standardized factor loadings and confidence intervals
cat("\nFACTOR LOADINGS FOR THE TWO-FACTOR MODEL\n")
cargas <- standardizedSolution(
  fit_2f,
  ci = TRUE
)

print(
  subset(
    cargas,
    op == "=~",
    select = c(lhs, rhs, est.std, se, ci.lower, ci.upper, pvalue)
  )
)

# Extract the correlation between the two factors
cat("\nCORRELATION BETWEEN TASK AND EGO\n")
print(
  subset(
    cargas,
    lhs == "Task" & op == "~~" & rhs == "Ego",
    select = c(lhs, op, rhs, est.std, ci.lower, ci.upper, pvalue)
  )
)

# Print the full summary of the two-factor model
cat("\nSUMMARY OF THE TWO-FACTOR MODEL\n")
summary(
  fit_2f,
  fit.measures = TRUE,
  standardized = TRUE,
  ci = TRUE
)
```

----

6. COMPOSITE RELIABILITY (CR) AND AVERAGE VARIANCE EXTRACTED (AVE)

CR and AVE were calculated from the standardized estimates of the correlated two-factor CFA model. The CFA was estimated using lavaan with ordinal indicators and the DWLS estimator.

```
library(lavaan)

# Define the CMI items
items <- paste0("I", 1:9)

# Specify the correlated two-factor CFA model
modelo_2f <- '
  Task =~ I1 + I3 + I5 + I6 + I7
  Ego  =~ I2 + I4 + I8 + I9
'

# Fit the CFA model
fit_2f <- cfa(
  model = modelo_2f,
  data = data,
  ordered = items,
  estimator = "DWLS",
  se = "robust",
  test = "scaled.shifted",
  parameterization = "delta"
)

# Extract standardized parameter estimates
sol <- standardizedSolution(fit_2f)

# Calculate CR and AVE for each factor
calcular_CR_AVE <- function(nombre_factor) {

  # Extract standardized factor loadings
  lambda <- sol$est.std[
    sol$op == "=~" & sol$lhs == nombre_factor
  ]

  # Identify the indicators for the factor
  indicadores <- sol$rhs[
    sol$op == "=~" & sol$lhs == nombre_factor
  ]

  # Extract standardized residual variances for the indicators
  error <- sol$est.std[
    sol$op == "~~" &
    sol$lhs == sol$rhs &
    sol$lhs %in% indicadores
  ]

  # Check that all loadings and residual variances were retrieved
  if (length(lambda) == 0 || length(error) != length(lambda)) {
    stop("Could not correctly extract loadings and residual variances for
", nombre_factor)
  }
```

```
  # Calculate composite reliability
  CR <- (sum(lambda)^2) /
    ((sum(lambda)^2) + sum(error))

  # Calculate average variance extracted
  AVE <- mean(lambda^2)

  return(c(CR = CR, AVE = AVE))
}

# Calculate results for both factors
resultados <- rbind(
  Task = calcular_CR_AVE("Task"),
  Ego  = calcular_CR_AVE("Ego")
)

# Display results to three decimal places
print(round(resultados, 3))
```

----
7. HETEROTRAIT-MONOTRAIT RATIO (HTMT) AND BOOTSTRAP CONFIDENCE INTERVAL

The HTMT ratio was calculated using polychoric correlations estimated with lavaan::lavCor(). Its 95% confidence interval was estimated using 5,000 bootstrap resamples with replacement.

```
library(lavaan)

# Define the items belonging to each factor
task_items <- c("I1", "I3", "I5", "I6", "I7")
ego_items  <- c("I2", "I4", "I8", "I9")
all_items <- c(task_items, ego_items)

# Check that all items are present in the dataset
stopifnot(all(all_items %in% names(data)))

# Calculate HTMT using polychoric correlations
calcular_htmt <- function(datos) {

  R <- lavCor(
    datos[, all_items, drop = FALSE],
    ordered = all_items
  )

  hetero <- abs(R[task_items, ego_items])

  task_cor <- abs(R[task_items, task_items])
  ego_cor  <- abs(R[ego_items, ego_items])

  task_cor <- task_cor[upper.tri(task_cor)]
  ego_cor  <- ego_cor[upper.tri(ego_cor)]

  mean(hetero) / sqrt(mean(task_cor) * mean(ego_cor))
}

# Calculate HTMT for the full sample
```

```
htmt_observado <- calcular_htmt(data)

cat("HTMT =", round(htmt_observado, 3), "\n")

# Perform 5,000 bootstrap resamples
set.seed(1234)
B <- 5000
n <- nrow(data)

htmt_boot <- replicate(B, {

  indices <- sample(seq_len(n), size = n, replace = TRUE)

  tryCatch(
    calcular_htmt(data[indices, , drop = FALSE]),
    error = function(e) NA_real_
  )
})

# Remove failed bootstrap estimates
htmt_boot <- htmt_boot[is.finite(htmt_boot)]

cat("Valid bootstrap resamples:", length(htmt_boot), "of", B, "\n")

# Calculate the 95% bootstrap confidence interval
if (length(htmt_boot) >= 4500) {

  ic <- quantile(
    htmt_boot,
    probs = c(0.025, 0.975),
    na.rm = TRUE
  )

  cat(
    "95% bootstrap CI: [",
    round(ic[1], 3), ", ",
    round(ic[2], 3), "]\n",
    sep = ""
  )

} else {
  cat("Warning:  More  than  500  bootstrap  resamples  failed.  Review  the
estimation.\n")
}
```
