# Supplementary material for: Initial Evaluation of the Internal Structure and Internal Consistency of the Context-Adapted Perceived Peer Motivational Climate Scale (CMI) Among Nursing Students Enrolled in a Clinical Simulation Program
Source: Behav Sci (Basel). 2026 Oct 17;16(10):1932. doi: 10.3390/bs16101932 (PMC13701605; doi:10.3390/bs16101932)
Supplement: Supplementary file 1 [file behavsci-16-01932-s001.zip › Supplementary_File_Tables and figures.pdf]

Supplementary Tables and Figures

Supplementary Table S1. Descriptive statistics and distributional characteristics of the nine items.

Item	Mean	SD	Median	IQR	Skewness	Kurtosis
1	3.07	1.11	3	2	0.171	−0.818
2	2.91	1.23	3	2	0.066	−1.000
3	3.26	1.10	3	2	−0.143	−0.855
4	2.95	1.22	3	2	0.028	−0.927
5	3.26	1.07	3	2	−0.021	−0.986
6	3.20	1.10	3	2	0.013	−0.920
7	3.20	1.07	3	2	0.059	−0.862
8	2.89	1.25	3	2	0.132	−1.030
9	2.91	1.23	3	2	0.047	−0.916

Note**.** $N = 395$ for all items; no missing observations. All items were scored from 1 to 5. Skewness and kurtosis refer to the distribution of the observed item scores; kurtosis is reported as excess kurtosis.

Supplementary Table S2. Pearson and polychoric correlation matrices among the nine items.

Panel A. Pearson correlations

Item	**1**	**2**	**3**	**4**	**5**	**6**	**7**	**8**	**9**
1	1.000	0.605	0.806	0.608	0.743	0.686	0.697	0.558	0.571
2		1.000	0.600	0.801	0.555	0.443	0.444	0.825	0.760
3			1.000	0.584	0.794	0.718	0.730	0.597	0.568
4				1.000	0.530	0.435	0.450	0.801	0.791
5					1.000	0.768	0.779	0.510	0.501
6						1.000	0.829	0.462	0.478
7							1.000	0.450	0.449
8								1.000	0.795
9									1.000

Panel B. Polychoric correlations

Item	**1**	**2**	**3**	**4**	**5**	**6**	**7**	**8**	**9**
1	1.000	0.645	0.868	0.654	0.798	0.735	0.749	0.594	0.615
2		1.000	0.642	0.846	0.604	0.480	0.482	0.875	0.811
3			1.000	0.621	0.852	0.775	0.788	0.638	0.612
4				1.000	0.572	0.470	0.487	0.850	0.841
5					1.000	0.831	0.843	0.553	0.542
6						1.000	0.894	0.494	0.521
7							1.000	0.480	0.486
8								1.000	0.848
9									1.000

Note. Pearson correlations are based on the observed item scores. Polychoric correlations were estimated from the ordinal response categories. $N = 395$. The polychoric correlation matrix was used for the primary exploratory factor analysis, whereas the Pearson correlation matrix was used for the maximum-likelihood sensitivity analysis.

Supplementary Table S3. Parallel analysis based on the Pearson correlation matrix for factor retention.

Factor	Observed eigenvalue	Simulated eigenvalue	Retained
1	5.69	0.51	Yes
2	1.02	0.17	Yes
3	0.02	0.12	No
4	−0.08	0.07	No
5	−0.11	0.02	No
6	−0.17	−0.02	No
7	−0.20	−0.07	No
8	−0.23	−0.12	No
9	−0.26	−0.18	No

Note. Parallel analysis was conducted using psych::fa.parallel based on the Pearson correlation matrix, with 1000 iterations and factor analysis (fa = "fa"). Factors were retained when the observed factor eigenvalue exceeded the corresponding simulated factor eigenvalue. The two-factor solution identified by parallel analysis was subsequently examined using the primary ordinal EFA based on the polychoric correlation matrix, minimum residual extraction, and oblimin rotation.

Supplementary Table S4. Polychoric parallel analysis for factor retention

Result	Factor 1	Factor 2	Factor 3
Observed factor eigenvalues	6.11	1.07	0.03
Simulated factor eigenvalues (95th percentile)	0.52	0.17	0.12

Note. Parallel analysis was conducted using polychoric correlations, minimum residual factor extraction, and 1000 iterations. The results supported the retention of two factors.

Supplementary Table S5. Exploratory factor analysis based on a polychoric correlation matrix, minimum residual extraction, and oblimin rotation.

Item	Task-involving factor	Ego-involving factor	Communality	Uniqueness
1	0.704	0.242	0.767	0.233
2	0.027	0.904	0.850	0.150
3	0.774	0.200	0.833	0.167
4	0.008	0.918	0.852	0.149
5	0.888	0.056	0.855	0.145
6	0.956	−0.077	0.827	0.173
7	0.991	−0.106	0.861	0.139
8	−0.019	0.947	0.875	0.125
9	0.032	0.876	0.803	0.197

Note. A polychoric correlation matrix, minimum residual extraction, and oblimin rotation were used. *N* = 395. The correlation between the two factors was *r* = 0.626. All pattern coefficients are shown.

Supplementary Table S6. Sensitivity exploratory factor analysis based on the Pearson correlation matrix, maximum likelihood extraction, and oblimin rotation.

Item	Task-involving factor	Ego-involving factor	Communality	Uniqueness
1	0.679	0.235	0.714	0.286
2	0.018	0.887	0.807	0.193
3	0.736	0.203	0.768	0.232
4	0.007	0.890	0.800	0.200
5	0.853	0.055	0.789	0.211
6	0.928	−0.079	0.777	0.223
7	0.952	−0.099	0.800	0.200
8	−0.011	0.914	0.822	0.178
9	0.029	0.848	0.750	0.250

Note: Maximum likelihood extraction with oblimin rotation based on the Pearson correlation matrix, $N = 395$. All pattern coefficients are shown without suppression. Communality represents the proportion of variance in each item accounted for by the retained factors; uniqueness = 1 − communality. The correlation between the two factors was $r = 0.621$.

Supplementary Table S7. Structure matrix of the sensitivity exploratory factor analysis

Item	Task-involving factor	Ego-involving factor
1	0.825	0.656
2	0.569	0.898
3	0.862	0.660
4	0.559	0.895
5	0.887	0.584
6	0.879	0.497
7	0.891	0.492
8	0.556	0.907
9	0.555	0.866

Note: Maximum likelihood extraction with oblimin rotation based on the Pearson correlation matrix, N = 395. The structure coefficients represent the correlations between the observed items and the rotated factors. The correlation between the two factors was $r = 0.621$.

Supplementary Figure S1. Parallel analysis based on Pearson correlations.

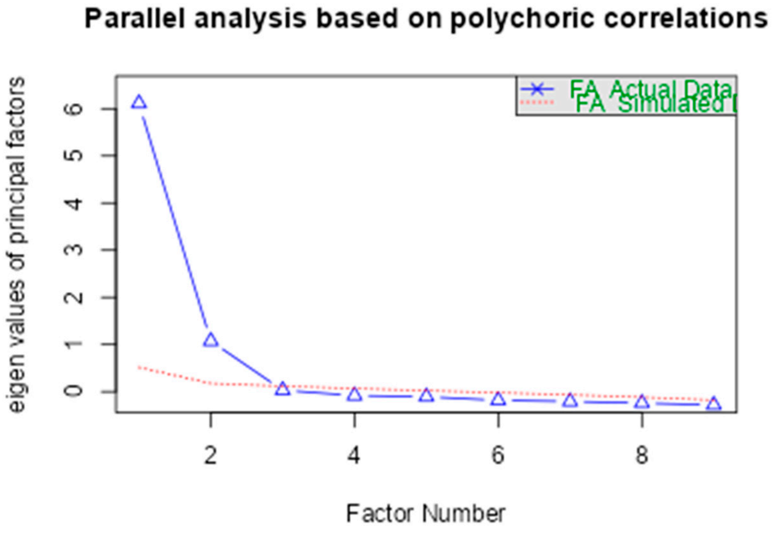

Supplementary Figure S2: Scree plot based on the polychoric correlation matrix.

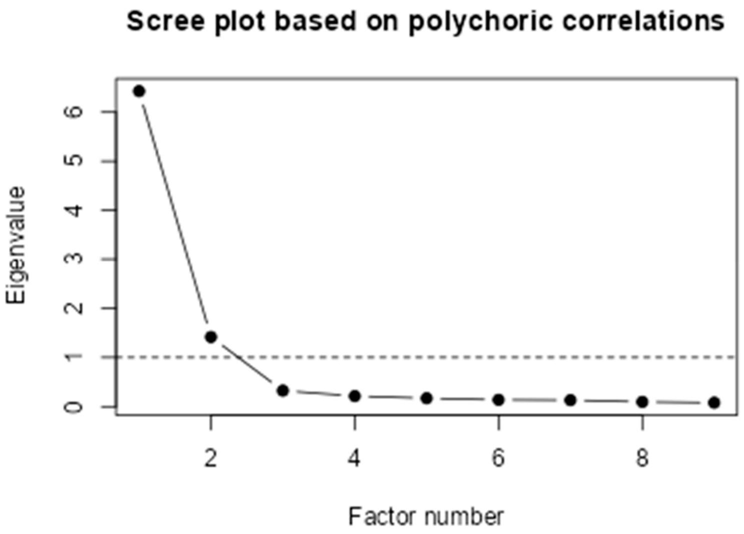

Supplementary Table S8. Complete CFA parameter estimates for the correlated two-factor model of the CMI

Parameter	Estimate	SE	z	*p*	95% CI	Std.
**Factor loadings**						
Task-involving factor						
1	1.000†	—	—	—	—	0.900
3	1.027	0.017	61.843	< 0.001	0.994 to 1.059	0.925
5	1.018	0.018	58.029	< 0.001	0.983 to 1.052	0.916
6	0.999	0.019	53.061	< 0.001	0.962 to 1.036	0.900
7	1.011	0.019	53.321	< 0.001	0.974 to 1.048	0.910
Ego-involving factor						
2	1.000†	—	—	—	—	0.924
4	0.995	0.019	53.661	< 0.001	0.958 to 1.031	0.919
8	1.014	0.018	56.264	< 0.001	0.978 to 1.049	0.936
9	0.975	0.018	55.471	< 0.001	0.941 to 1.009	0.901
**Ordinal thresholds**						
1 τ1	−1.155	0.081	−14.240	< 0.001	−1.314 to −0.996	—
1 τ2	−0.394	0.065	−6.064	< 0.001	−0.521 to −0.267	—
1 τ3	0.387	0.065	5.964	< 0.001	0.260 to 0.514	—
1 τ4	1.592	0.103	15.481	< 0.001	1.390 to 1.794	—
3 τ1	−1.119	0.080	−14.014	< 0.001	−1.275 to −0.962	—
3 τ2	−0.105	0.063	−1.658	0.097	−0.229 to 0.019	—
3 τ3	0.580	0.067	8.634	< 0.001	0.448 to 0.711	—
3 τ4	1.689	0.110	15.396	< 0.001	1.474 to 1.904	—
5 τ1	−1.143	0.081	−14.166	< 0.001	−1.301 to −0.985	—
5 τ2	−0.124	0.063	−1.959	0.050	−0.248 to 0.000	—
5 τ3	0.535	0.067	8.045	< 0.001	0.405 to 0.666	—
5 τ4	1.955	0.134	14.588	< 0.001	1.692 to 2.217	—
6 τ1	−1.119	0.080	−14.014	< 0.001	−1.275 to −0.962	—
6 τ2	−0.208	0.064	−3.264	0.001	−0.332 to −0.083	—
6 τ3	0.499	0.066	7.552	< 0.001	0.370 to 0.629	—
6 τ4	1.745	0.114	15.294	< 0.001	1.521 to 1.968	—
7 τ1	−1.143	0.081	−14.166	< 0.001	−1.301 to −0.985	—

Parameter	Estimate	SE	z	*p*	95% CI	Std.
7 τ2	−0.247	0.064	−3.866	< 0.001	−0.372 to −0.122	—
7 τ3	0.535	0.067	8.045	< 0.001	0.405 to 0.666	—
7 τ4	1.840	0.122	15.035	< 0.001	1.600 to 2.079	—
2 τ1	−1.219	0.084	−14.594	< 0.001	−1.383 to −1.055	—
2 τ2	−0.387	0.065	−5.964	< 0.001	−0.514 to −0.260	—
2 τ3	0.240	0.064	3.765	< 0.001	0.115 to 0.365	—
2 τ4	1.072	0.078	13.699	< 0.001	0.919 to 1.226	—
4 τ1	−1.180	0.082	−14.386	< 0.001	−1.341 to −1.019	—
4 τ2	−0.401	0.065	−6.164	< 0.001	−0.528 to −0.273	—
4 τ3	0.326	0.064	5.066	< 0.001	0.200 to 0.452	—
4 τ4	1.095	0.079	13.858	< 0.001	0.940 to 1.250	—
8 τ1	−1.167	0.082	−14.314	< 0.001	−1.327 to −1.008	—
8 τ2	−0.408	0.065	−6.263	< 0.001	−0.535 to −0.280	—
8 τ3	0.188	0.064	2.963	0.003	0.064 to 0.313	—
8 τ4	1.061	0.078	13.618	< 0.001	0.908 to 1.214	—
9 τ1	−1.180	0.082	−14.386	< 0.001	−1.341 to −1.019	—
9 τ2	−0.456	0.066	−6.958	< 0.001	−0.585 to −0.328	—
9 τ3	0.319	0.064	4.966	< 0.001	0.193 to 0.446	—
9 τ4	1.007	0.076	13.203	< 0.001	0.858 to 1.157	—
**Residual variances**						
1	0.189	—	—	—	—	0.189
3	0.145	—	—	—	—	0.145
5	0.161	—	—	—	—	0.161
6	0.191	—	—	—	—	0.191
7	0.172	—	—	—	—	0.172
2	0.147	—	—	—	—	0.147
4	0.156	—	—	—	—	0.156
8	0.123	—	—	—	—	0.123
9	0.189	—	—	—	—	0.189
**Latent factor variances**						
Task-involving	0.811	0.024	33.259	< 0.001	0.763 to 0.858	1.000
Ego-involving	0.853	0.023	37.392	< 0.001	0.808 to 0.898	1.000

Parameter	Estimate	SE	z	*p*	95% CI	Std.
**Latent factor covariance**						
Task-involving↔ Ego-involving	0.567	0.029	19.367	< 0.001	0.509 to 0.624	0.681

Note. SE = standard error; CI = confidence interval; Std. = standardized estimate; τ = ordinal threshold. The CFA was estimated using the diagonally weighted least squares (DWLS) estimator with robust standard errors and a mean-adjusted scaled-and-shifted test statistic, treating the nine CMI items as ordered categorical variables. The first loading of each latent factor was fixed to 1 for model identification. The model converged successfully after 36 iterations. The standardized latent factor correlation was r = 0.681 (p < 0.001), with a 95% CI of 0.623 to 0.739. The confidence interval of 0.509 to 0.624 shown in the table refers to the unstandardized latent covariance.

Supplementary Table S9. Model-based psychometric indicators of the correlated two-factor CFA model

Indicator	Value
CR — Task-involving	0.960
CR — Ego-involving	0.957
AVE — Task-involving	0.828
AVE — Ego-involving	0.846
HTMT — Task-involving vs. Ego-involving	0.675, 95% CI 0.613 to 0.730

Note. CR = composite reliability; AVE = average variance extracted; HTMT = heterotrait–monotrait ratio. CR and AVE were calculated from the standardized factor loadings of the correlated two-factor CFA model. HTMT was estimated from the item data. These indices are reported as model-based psychometric information and should not be interpreted as establishing convergent or discriminant validity on their own.
